# Lithium Nuclear Spin Polarization Lifetimes as Sensitive Reporters of Battery Electrolyte Degradation

**DOI:** 10.1021/acsomega.6c04272

**Published:** 2026-05-14

**Authors:** Florin Teleanu, Zhiyuan Gao, Alexej Jerschow

**Affiliations:** † Department of Chemistry, 5894 New York University, New York, New York 10003, United States; ‡ ELI-NP, “Horia Hulubei” National Institute for Physics and Nuclear Engineering, 30 Reactorului Street, Bucharest-Magurele, Ilfov 077125, Romania

## Abstract

Nondestructive monitoring of electrolyte degradation is a critical, yet unresolved challenge in the diagnostics of lithium-ion batteries. Here, we report the potential utility of nuclear spin polarization lifetime T_1_ as a highly sensitive, intrinsic probe for monitoring the health of a model LP30 electrolyte under thermal cycling by using multinuclear NMR spectroscopy. We identified a particularly sensitive correlation between the degradation state and ^7^Li relaxation dynamics that is not observed for other relevant nuclei. Molecular dynamics simulations provide a mechanistic rationale, linking the observed relaxation changes to specific alterations in the Li-ion solvation shell structure during the degradation. These findings establish ^7^Li spin lifetime as a robust, field-independent analytical metric, with potential application to the noninvasive assessment of battery state-of-health in regimes where chemical shift resolution is insufficient for species discrimination.

## Graphical abstract

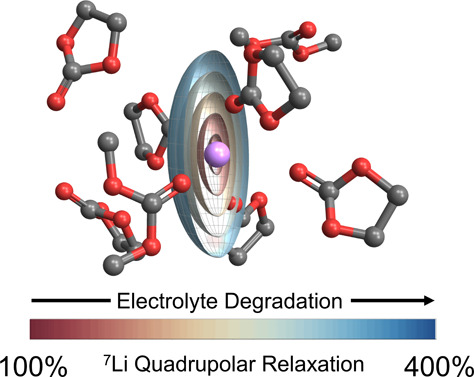

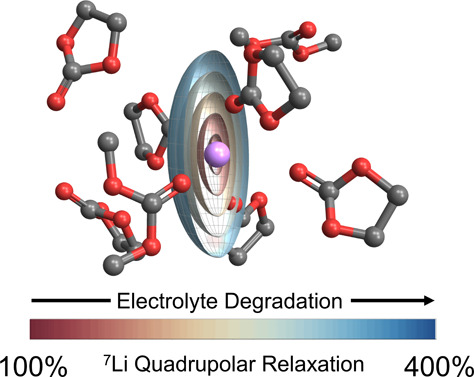

## Introduction

Lithium-ion batteries (LIBs) have become indispensable for energy storage, powering portable electronics, and electric vehicles.^1^ LiPF_6_ dissolved in carbonate-based solvents is one of the most widely used electrolytes due to its high conductivity, solubility, and ability to facilitate the formation of a stable solid electrolyte interphase (SEI).^2^ The SEI is generated by LiPF_6_ degradation to form various lithium salts,^3^ which, in part, can further precipitate and contribute to the SEI formation on the graphite anode.^4^ The structured SEI ensures reversible lithium intercalation, which sustains hundreds of charge–discharge cycles by restricting electron tunneling and preventing electrolyte reduction,^5,6^ thereby enhancing battery reliability and longevity.^7^

A common carbonate solvent mixture for LiPF_6_ is made from ethylene carbonate (EC) and dimethyl carbonate (DMC),^8^ a system referred to as LP30. EC facilitates the formation of a stable SEI layer on graphite anodes.^9^ In addition, its high dielectric constant allows efficient dissolution of the LiPF_6_ salt, thereby supporting good ionic conductivity,^10^ while its thermal stability contributes to the safety of the LIBs. On the other hand, DMC offers a low viscosity, which enhances electrolyte conductivity. Furthermore, EC and DMC are widely recognized as cost-effective organic solvents. Therefore, the combined use of EC and DMC achieves a very good balance between SEI stability and ionic conductivity, which explains their wide use as a binary solvent system in LIBs.^11^

Since LIBs are omnipresent nowadays, there is an urgent need to monitor the extent of degradation, ideally during thousands of charge–discharge cycles in a nondestructive way. Common noninvasive techniques that allow for *in situ* ^12^ and *operando* ^13^ measurements include electrochemical impedance spectroscopy (EIS),^14^ X-ray computed tomography (XCT),^15–17^ ultrasonic testing (UT),^18^ infrared thermography (IRT),^19^ nuclear magnetic resonance (NMR) spectroscopy,^20,21^ Raman spectroscopy,^22^ and other spectroscopic methods.^23,24^ Among these, NMR spectroscopy provides distinct advantages as a nondestructive diagnostic method for monitoring battery components with atomic resolution. In contrast to conventional electrochemical or imaging approaches, NMR can directly probe the local chemical and dynamic environments of specific nuclei (e.g., ^1^H, ^7^Li, ^19^F, ^31^P) within electrolytes.^25^ This capability enables simultaneous investigations of ion transport, solvation structures, relaxation dynamics, and chemical degradation pathways without the need to disassemble or alter the cells. The nondestructive nature of NMR further allows for *in situ* ^20^ measurements, thereby providing insights into the electrolyte behavior under practical cycling conditions. More recently, it was demonstrated that NMR transitions can be excited and detected through the metallic casings of realistic batteries in zero-to-ultralow field setups using optical magnetometers for signal acquisition.^26^

NMR relaxation and diffusion measurements are highly sensitive to subtle variations in molecular mobility and electrolyte composition, thereby potentially enabling the detection of early-stage degradation phenomena and latent defects that might remain undetected by macroscopic characterization methods. The spin polarization lifetime of quadrupolar nuclei such as ^7^Li (I = 3/2) in battery electrolytes is mostly governed by the quadrupolar interaction.^27^ The dynamic solvation environment of Li^+^ modulates the electric field gradients (EFGs) around the nucleus as solvent molecules or ions bind transiently. For spin-1/2 nuclei, such as ^1^H, ^19^F and ^31^P, relaxation is governed by intra- and intermolecular dipolar interactions and chemical shift anisotropy^28^ modulated by the rotational and translational tumbling of molecules. In addition, the presence of paramagnetic impurities, for example, transition metal ions leached from electrodes or oxygen dissolved into electrolytes, can greatly accelerate the relaxation of all nuclei due to strong dipolar coupling even within μM concentrations.^29,30^ Here, we show how electrolyte degradation leads to a significant enhancement of relaxation rates for ^7^Li in a controlled and chemically simplified LP30 model system, with only minor increases for the other spin-1/2 nuclei. Molecular dynamics (MD) simulations are employed to identify a possible mechanistic rationale for the observed effects by predicting ^7^Li polarization lifetimes in agreement with the experimental trend. Our findings demonstrate the analytical power of the ^7^Li polarization lifetime as a sensitive indicator of electrolyte health that can be measured with standard NMR methods, with promising advantages for noninvasive evaluation through the metallic housings of commercial cells in regimes where chemical shift resolution is insufficient.^26^

## Experimental Methods

### Sample Preparation

A 1.0 M lithium hexafluorophosphate (LiPF_6_) solution in a 1:1 (v/v) mixture of ethylene carbonate (EC) and dimethyl carbonate (DMC), battery grade, was purchased from Sigma-Aldrich (Cat. No. 746711). The solution was transferred into the inner compartment of a coaxial NMR tube (1.96 mm inner diameter) inside an argon-filled glovebox. Deuterated dimethyl sulfoxide (DMSO-d6) was placed in the outer compartment to serve as a magnetic field lock. Immediately after preparation in the glovebox, the sample was sealed with Parafilm and subsequently placed in a water bath at 55 °C inside a fume hood to induce systematic degradation. Samples were allowed to cool at room temperature where all measurements were performed to ensure consistency. Then, the samples were placed back into the water bath for further degradation and measurements for a maximum total heating duration of 300 h. A total of three identical samples were prepared on different dates, all of which exhibited highly consistent results, confirming the reproducibility of the experimental protocol.

### NMR Experiments

All NMR experiments were performed with a Bruker Avance III 400 NMR spectrometer at 298 K. For all ^1^H, ^7^Li, and ^19^F nuclei, we measured the longitudinal relaxation rates using the inversion recovery pulse sequence (T1IR).

### Molecular Dynamics Simulations

All simulations were performed with GROMACS 2023 ^31^ for systems containing 1 M LiPF6 ions in EC:DMC mixtures with different molar ratios of the two solvents (See Tables S1 and S2 for all compositions). The united-atoms TraPPE force field^32^ was used throughout, as it was proven to predict more accurate transport properties compared to all-atom force fields such as OPLS and CHARMM. Energy minimization was followed by 0.5 ns NPT equilibration with the Berendsen barostat^33^ with a time constant of 2 ps and a compressibility of 10^–4^ bar^–1^. A 10 ns production run was performed under NPT conditions using a Parrinello–Rahman barostat^34^ with a time constant of 5 ps. The number of molecules was chosen to target the 1 M concentration of ions and the relative ratios of the two solvent components as seen from experiments. We emulated the creation of carbonate ions by adding ${\mathrm{CO}}_{3}^{2-}$ species as the DMC concentration was reduced. For each added carbonate anion, two hexafluorophosphate molecules were eliminated in order to preserve charge neutrality. The simulation box size was 8 nm, and the system used periodic boundary conditions with the Ewald summation for long-range electrostatics up to 14 Å. All reported properties were derived after averaging over 5 independent runs starting from different initial boxes created using Packmol.^35^

Relaxation rate predictions from MD simulations were performed by calculating quadrupolar coupling correlation functions according to the procedures described in previous work with a custom script.^27,36^ The time-dependent components of the electric field gradient (EFG) tensor are computed for each Li ion in consecutive MD frames. The local instantaneous contribution to the EFG tensor of a targeted Li ion by an atom X characterized by a partial charge *q*_X_ at distance *r*_LiX_ is given by

1 $$V_{Li}^{\alpha \beta }\left({\mathrm{r⃗}}_{LiX}\right)=\left(1+\gamma_{Li}\right)\frac{q_{X}}{4\pi \epsilon_{0}r_{LiX}^{3}}\left\{\frac{3r_{LiX}^{\alpha }:r_{LiX}^{\beta }}{r_{LiX}^{2}}-\delta_{\alpha \beta }\right\}$$

where $r_{LiX}^{\mu }$ are components of the interatomic vector *r⃗*_LiX_ between the central lithium ion and the neighboring atom X, ϵ_0_ is the vacuum magnetic permeability, δ_αβ_ is the Kronecker delta function, *q*_X_ is the effective atomic charge of atom X, and γ_Li_ is the Sternheimer antishielding factor accounting for the core electron polarization of the lithium ion.^37^ We focused only on relative changes of predicted ^7^Li R_1_ rates as quantitative predictions would require either highly expensive *ab initio* simulations^38^ or accurate calculation of the Sternheimer factor^39^ which are beyond the scope of this paper.

The autocorrelation function of the ensemble-averaged time-dependent EFG tensor over all Li ions *N*_Li_ in the simulation box is calculated using

2 $$G\left(t\right)=\frac{1}{N_{Li}}\overset{N_{Li}}{\underset{1}{\sum }}\overset{N_{X}}{\underset{1}{\sum }}\frac{1}{6}\overset{3}{\underset{\alpha =1}{\sum }}\overset{3}{\underset{\beta =1}{\sum }}\left\langle V_{LiX}^{\alpha \beta }\left(0\right)\cdot V_{LiX}^{\alpha \beta }\left(t\right)\right\rangle$$

where we considered the contributions of all *N*_X_ atoms within a cutoff distance of 10 Å around individual Li ions. The correlation time of the fluctuating quadrupolar is computed as the integral of the normalized autocorrelation function *G*(*t*) via $\tau_{C}=G{\left(0\right)}^{-1}\int_{0}^{\infty }dtG\left(t\right)$ . The autorelaxation rate of ^7^Li nuclei is thus computed as^27^

3 $$R_{1}^{7Li}=\frac{2S+3}{20S^{2}\left(2S-1\right)}{\left(\frac{eQ}{\hbar }\right)}^{2}\left\langle V^{2}\right\rangle \tau_{C}$$

where ⟨**V** ^2^⟩ is the variance of the ensemble-averaged EFG tensor, *e* is the elementary charge, *ℏ* is the reduced Planck constant, and *S* and *Q* are the nuclear spin and quadrupole moment of the ^7^Li nucleus.

## Results and Discussion

Sequential 1D NMR spectra of multiple nuclei were acquired for a standard sample of 1 M LiPF_6_ dissolved in EC:DMC = 1:1 (v:v) undergoing degradation via thermal cycling (See Experimental Methods). The most striking change upon degradation was observed for the relative ratio of the two solvent components [DMC]:[EC]. Initially, this value starts from 0.78 (Figure 1A) and more than halves through the explored degradation levels as DMC is more prone to decomposition into new chemical species such as carbonate anions.^40–43^ We chose this intrinsic and simple ^1^H spectral feature (decreasing [DMC]:[EC] ratio) as the principal reporter of solvent degradation in order to eliminate any variability/errors regarding the exact duration and temperature at which thermal cycling was performed. Additionally, we explored other NMR-active nuclei such as ^7^Li (Figure 1B), and ^19^F and ^31^P (Figure 1C,D) to identify ${\mathrm{PF}}_{6}^{-}$ oxidation byproducts, clearly showing the appearance of degradation products OPF­(OH)_2_, OPF_2_(OH), OPF­(OMe)­(OH), and OPF_2_(OMe), as identified in previous studies.^40,44,45^ Notably, the spectral features mentioned above are only accessible in high-field NMR spectroscopy which provides chemical shift resolution. However, the same experimental conditions prohibit the use of commercial batteries within metallic casings due to skin depth limitations, Eddy currents, and susceptibility issues.^21,46^ It is desirable to identify a new field-independent reporter of electrolyte health that overcomes these challenges as NMR detection has developed beyond standard induction coils and can now be performed with highly sensitive quantum magnetometers at ultralow fields ranging from nT to μT^47^, skin depth limitations are significantly reduced or eliminated, albeit with more spectral complexity and crowding.^26^

**Figure 1:**
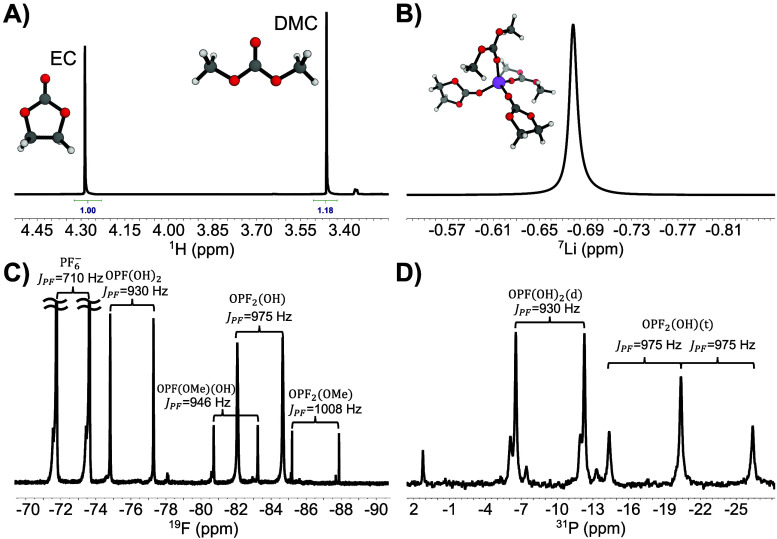
Multinuclear magnetic resonance spectroscopy analysis of 1 M LiPF_6_ in EC:DMC solvent mixture before and after degradation. (A) ^1^H spectrum of a fresh sample corresponding to EC:DMC = 1:1 (v/v). The ^1^H integral ratio is 1:1.18 which translates to an initial concentration ratio of the two solvent components of [DMC]:[EC] = 0.78. (B) ^7^Li spectrum of a fresh sample showing a single Lorentzian peak (Inset: a graphical representation of a transient Li^+^ solvation environment). (C) ^19^F spectrum of a thermally degraded sample revealing multiple byproducts of ${\mathrm{PF}}_{6}^{-}$ oxidation. (D) ^31^P spectrum confirming the accurate identification of the most abundant degradation products.

We examine now how NMR relaxation rates change upon thermal stress and how they correlate with the chosen degradation marker ([DMC]:[EC] ratio). Longitudinal polarization lifetimes characterize the recovery of nuclear spin populations at thermal equilibrium upon perturbations induced by radio frequency pulses. Generally, the relaxation rate constant R_1_ describing this process is field-independent (in the fast-tumbling regime),^28^ it is not affected by field inhomogeneities and can be independently measured for distinct nuclei with different gyromagnetic ratios. In our experiments, we observe a highly sensitive correlation of the ^7^Li polarization lifetime with the degradation stage of the solvent mixture (Figure 2A). The relative increase of ^7^Li R_1_ is more than 350% over the observed range of the [DMC]:[EC] reporter values, while other relaxation rates for spin-1/2 nuclei are only modestly increasing (≤50%). These trends further correlate with the increasing concentration of salt byproducts (Figure 2B), making ^7^Li R_1_ a promising reporter of the overall health of electrolytes. The differential impact on the relaxation rates of spin-1/2 (^1^H and ^19^F) and quadrupolar (^7^Li) nuclei suggests that the observed effects cannot be explained by (i) an overall increase in the sample’s viscosity^48^ which would have led to slower tumbling rates for all nuclei to a similar extent or (ii) intermolecular electronic dipolar contributions from possible paramagnetic species^29^ as nuclei with higher gyromagnetic ratios should have shown the largest increase in relaxation rates.

**Figure 2:**
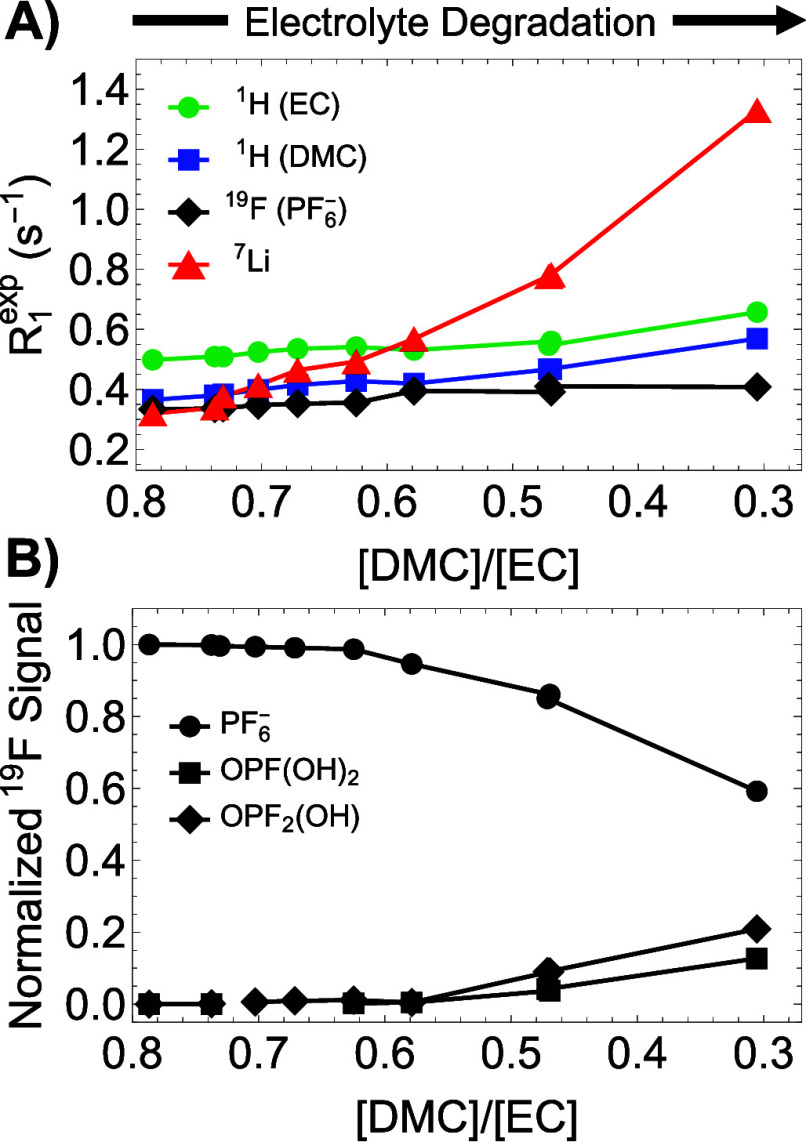
(A) Experimental longitudinal relaxation rates of multiple nuclei corresponding to both salt (^7^Li and ^19^F) and solvent (^1^H) components plotted as a function of the measured [DMC]/[EC] solvent ratio at different time points during the degradation process. (B) Correlation of the relative concentration of fluorophosphate species (quantified using peak integrals from ^19^F spectra) and the measured solvent ratio upon degradation. The data reflect three independent thermal-degradation measurements, each conducted on a different fresh sample and monitored over distinct time intervals.

To obtain an in-depth understanding of the mechanisms leading to enhanced ^7^Li quadrupolar relaxation, we performed MD simulations on systems with emulated electrolyte degradation stages and predicted the ^7^Li relaxation rates in an attempt to replicate the observed experimental trend (See Experimental Methods). Previous studies explored the changes of static and transport properties of the electrolyte in carbonate systems with variable solvent mixtures.^32,49^ However, by manually tuning in our simulations the [DMC]:[EC] ratios to similar values as the ones measured in the experiments, the predicted enhancement of ^7^Li R_1_ was insignificant (Figure 3F, black circles), despite significant changes in Li’s first solvation shell and the self-diffusion rates of all species (See Figure S1). Inspired by previous computational studies of potential degradation mechanisms,^3^ we turned our attention to carbonate ions produced during solvent degradation^40^ which are expected to bind Li^+^ ions more tightly than neutral carbonate molecules. Limited by the lack of adequate reactive force fields that can simulate bond breaking during degradation, we opted for a simple case study: as the [DMC]:[EC] solvent ratio decreased in our simulation boxes, we added one carbonate ion ${\mathrm{CO}}_{3}^{2-}$ for every 20 dimethyl carbonate (DMC) molecules removed from the system (Figure 3). The stronger binding of ${\mathrm{CO}}_{3}^{2-}$ to lithium ions is reflected by the radial distribution functions (Figure 3B), despite its negligible overall contribution to Li’s surroundings (Figure 3C). Thus, the carbonate anion is more effective in inducing a longer-lived EFG interaction as shown by its significantly larger residence time in Li’s first solvation shell (Figure 3D). At the same time, the predicted self-diffusion rates still follow experimental trends reported for different ratios of [DMC]:[EC] solvents^40,48^ despite the additional highly charged species added to our simulation boxes (Figure 3E). These cumulative effects increase the quadrupolar interaction experienced by ^7^Li nuclei, replicating well our experimental R_1_ trend (Figure 3F, red squares). To further confirm our hypothesis, we performed similar simulations with two times more carbonate ions and obtained nearly doubled ^7^Li relaxation rates (Figure 3F, red rhomboids). Our choice of incorporating carbonate anions ${\mathrm{CO}}_{3}^{2-}$ is not unique, as several other organic monocarbonates were proved to accumulate during degradation^40,50^ as well as ethoxy salts or poly­(ethylene oxide) fragments. However, a similar effect is expected regardless of the nature of the anion, as these negatively charged oxygen-bearing species are expected to bind stronger than solvent carbonates and induce larger quadrupolar interactions.

**Figure 3:**
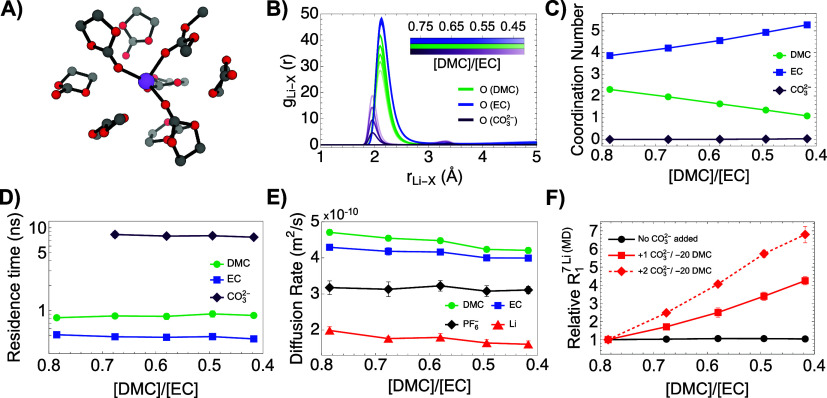
Molecular dynamics analysis of changes in the Li^+^ coordination shell vs [DMC]:[EC] ratios with increasing number of carbonate ions (one carbonate ion ${\mathrm{CO}}_{3}^{2-}$ was introduced for every 20 dimethyl carbonate (DMC) molecules removed from the system). (A) MD snapshot of the Li^+^ coordination shell up to 10 Å. (B) Radial distribution function of selected atoms around lithium ions (*g*_Li-X_). (C) Coordination number of selected species around lithium ions. (D) Residence time of selected species in the first solvation shell (≤3 Å) of lithium ions. (E) Diffusion rates of electrolyte components vs [DMC]:[EC] solvent ratio. (F) Relative increase in MD-predicted ^7^Li R_1_ values as a function of solvent composition with (red symbols) or without (black circles) added carbonate ions. All reported properties were derived after averaging over five independent runs (error bars shown).

## Conclusions

Nondestructive battery diagnostics based on low- and zero-to-ultralow-field (ZULF) detection of NMR signals from electrolytes in batteries could become powerful techniques for assessing and monitoring the electrolyte aging and composition over time.^26^ The limitation of low-field NMR is the lack of spectral dispersion that would allow for identifying different species. We describe herein experiments showing how NMR relaxation rates change with the thermal degradation of a common lithium-ion battery electrolyte mixture (LP30). We find that ^7^Li relaxation rates increase much more strongly with the degradation level, while other nuclei respond in a much weaker fashion. Using MD simulations, we provide a mechanistic rationale for the extraordinary sensitivity of ^7^Li R_1_ to the chemical environment following solvent degradation. On the basis of our findings, it should therefore be possible to use ^7^Li polarization lifetime as a reporter of electrolyte degradation. These health reporters could be measured with low-cost benchtop, earth-field, or ultralow-field setups that use magnetometer-based detectors.

## Supplementary Material



## Notes

Composition of MD simulation boxes; Comparative analysis of simulated MD scenarios (PDF)

## References

[1] Li M., Lu J., Chen Z., Amine K. (2018). 30 Years of Lithium-Ion Batteries. Adv. Mater.

[2] Aoki Y., Oda M., Kojima S., Yamaga Y., Ishihama T., Nagashima T., Doi T., Inaba M. (2023). Effective Approach by Computational Chemical Prediction
and Experimental Verification to Elucidate SEI Formation Mechanism
in LiPF6 -, LiFSI-, and LiBF4 -Containing Electrolyte Solutions. J. Phys. Chem. C.

[3] Spotte-Smith E. W. C., Petrocelli T. B., Patel H. D., Blau S. M., Persson K. A. (2023). Elementary Decomposition Mechanisms of Lithium Hexafluorophosphate
in Battery Electrolytes and Interphases. ACS
Energy Lett..

[4] Li J., Hu X., Li T. (2024). Simulation of Solid Electrolyte Interphase Growth for
Lithium Batteries Based on Kinetic Monte Carlo. Energy Mater. Adv..

[5] Wang A., Kadam S., Li H., Shi S., Qi Y. (2018). Review on
modeling of the anode solid electrolyte interphase (SEI) for lithium-ion
batteries. Npj Comput. Mater..

[6] Adenusi H., Chass G. A., Passerini S., Tian K. V., Chen G. (2023). Lithium Batteries
and the Solid Electrolyte Interphase (SEI)—Progress and Outlook. Adv. Energy Mater..

[7] Zhang S., Li Y., Bannenberg L. J., Liu M., Ganapathy S., Wagemaker M. (2024). The lasting impact of formation cycling
on the Li-ion
kinetics between SEI and the Li-metal anode and its correlation with
efficiency. Sci. Adv..

[8] Liao Z., Zhang S., Zhao Y., Qiu Z., Li K., Han D., Zhang G., Habetler T. G. (2020). Experimental evaluation of thermolysis-driven
gas emissions from LiPF6-carbonate electrolyte used in lithium-ion
batteries. J. Energy Chem..

[9] Lundström R., Gogoi N., Melin T., Berg E. J. (2024). Unveiling Reaction
Pathways of Ethylene Carbonate and Vinylene Carbonate in Li-Ion Batteries. J. Phys. Chem. C.

[10] Kang G., Zhong G., Ma J., Yin R., Cai K., Jia T., Ren X., Yu K., Qin P., Chen Z., Kang F., Cao Y. (2022). Weakly solvated EC-free
linear alkyl
carbonate electrolytes for Ni-rich cathode in rechargeable lithium
battery. iScience.

[11] Plichta E. J., Behl W. K. (2000). A low-temperature electrolyte for
lithium and lithium-ion
batteries. J. Power Sources.

[12] Lin X., Shen Y., Yu Y., Huang Y. (2024). In Situ NMR Verification
for Stacking Pressure-Induced Lithium Deposition and Dead Lithium
in Anode-Free Lithium Metal Batteries. Adv.
Energy Mater..

[13] Huang J., Albero Blanquer L., Bonefacino J., Logan E. R., Alves
Dalla Corte D., Delacourt C., Gallant B. M., Boles S. T., Dahn J. R., Tam H.-Y., Tarascon J.-M. (2020). Operando decoding
of chemical and thermal events in commercial Na­(Li)-ion cells via
optical sensors. Nature Energy.

[14] Zhang R. (2018). A Study on the Open
Circuit Voltage and State of Charge Characterization
of High Capacity Lithium-Ion Battery Under Different Temperature. Energies.

[15] Li L., Hou J. (2018). Capacity detection
of electric vehicle lithium-ion batteries based
on X-ray computed tomography. RSC Adv..

[16] Ho A. S., Parkinson D. Y., Finegan D. P., Trask S. E., Jansen A. N., Tong W., Balsara N. P. (2021). 3D Detection of Lithiation and Lithium
Plating in Graphite Anodes during Fast Charging. ACS Nano.

[17] Dayani S., Markötter H., Schmidt A., Widjaja M. P., Bruno G. (2023). Multi-level
X-ray computed tomography (XCT) investigations of commercial lithium-ion
batteries from cell to particle level. J. Energy
Chem..

[18] Deng Z., Huang Z., Shen Y., Huang Y., Ding H., Luscombe A., Johnson M., Harlow J. E., Gauthier R., Dahn J. R. (2020). Ultrasonic Scanning
to Observe Wetting and “Unwetting”
in Li-Ion Pouch Cells. Joule.

[19] Marino C., Boulaoued A., Fullenwarth J., Maurin D., Louvain N., Bantignies J.-L., Stievano L., Monconduit L. (2017). Solvation
and Dynamics of Lithium Ions in Carbonate-Based Electrolytes during
Cycling Followed by Operando Infrared Spectroscopy: The Example of
NiSb2, a Typical Negative Conversion-Type Electrode Material for Lithium
Batteries. J. Phys. Chem. C.

[20] Gunnarsdóttir A. B., Amanchukwu C. V., Menkin S., Grey C. P. (2020). Noninvasive In Situ
NMR Study of “Dead Lithium” Formation and Lithium Corrosion
in Full-Cell Lithium Metal Batteries. J. Am.
Chem. Soc..

[21] Pecher O., Carretero-González J., Griffith K. J., Grey C. P. (2017). Materials’
Methods: NMR in Battery Research. Chem. Mater..

[22] Bouteau G., Van-Nhien A. N., Sliwa M., Sergent N., Lepretre J.-C., Gachot G., Sagaidak I., Sauvage F. (2019). Effect of standard
light illumination on electrolyte’s stability of lithium-ion
batteries based on ethylene and di-methyl carbonates. Sci. Rep..

[23] Gervillié-Mouravieff C., Bao W., Steingart D. A., Meng Y. S. (2024). Non-destructive characterization
techniques for battery performance and life-cycle assessment. Nat. Rev. Electr. Eng..

[24] Chacón X. C. A., Laureti S., Ricci M., Cappuccino G. (2023). A Review of
Non-Destructive Techniques for Lithium-Ion Battery Performance Analysis. World Electr. Veh. J..

[25] Shi Z., Guo D., Canlas C. G., Zhu Y., Alshareef H. N. (2025). Liquid-State
NMR Spectroscopy for Battery Electrolyte Design. ACS Energy Lett..

[26] Fabricant A. M. (2026). Enabling nondestructive observation of electrolyte
composition in
batteries with ultralow-field nuclear magnetic resonance. Chem. Sci..

[27] Mohammadi M., Benders S., Jerschow A. (2020). Nuclear magnetic resonance spin-lattice
relaxation of lithium ions in aqueous solution by NMR and molecular
dynamics. J. Chem. Phys..

[28] Kowalewski, J. ; Maler, L. Nuclear Spin Relaxation in Liquids: theory, Experiments, and Applications, 2nd ed.; CRC Press LLC, 2019.

[29] Allen J. P., Grey C. P. (2023). Solution NMR of
Battery Electrolytes: Assessing and
Mitigating Spectral Broadening Caused by Transition Metal Dissolution. J. Phys. Chem. C.

[30] Kruk D., Kowalewski J. (2009). General treatment of paramagnetic
relaxation enhancement
associated with translational diffusion. J.
Chem. Phys..

[31] Abraham M. J., Murtola T., Schulz R., Páll S., Smith J. C., Hess B., Lindahl E. (2015). GROMACS: High performance
molecular simulations through multi-level parallelism from laptops
to supercomputers. SoftwareX.

[32] Luo Z., Burrows S. A., Smoukov S. K., Fan X., Boek E. S. (2023). Extension
of the TraPPE Force Field for Battery Electrolyte Solvents. J. Phys. Chem. B.

[33] Berendsen H. J. C., Postma J. P. M., van Gunsteren W. F., DiNola A., Haak J. R. (1984). Molecular
dynamics with coupling to an external bath. J. Chem. Phys..

[34] Parrinello M., Rahman A. (1981). Polymorphic transitions in single crystals: A new molecular
dynamics method. J. Appl. Phys..

[35] Martínez L., Andrade R., Birgin E. G., Martínez J. M. (2009). PACKMOL:
A package for building initial configurations for molecular dynamics
simulations. J. Comput. Chem..

[36] Chubak I., Alon L., Silletta E. V., Madelin G., Jerschow A., Rotenberg B. (2023). Quadrupolar
23Na+ NMR relaxation as a probe of subpicosecond
collective dynamics in aqueous electrolyte solutions. Nat. Commun..

[37] Sternheimer R. M. (1966). Shielding
and Antishielding Effects for Various Ions and Atomic Systems. Phys. Rev..

[38] Badu S., Truflandier L., Autschbach J. (2013). Quadrupolar NMR Spin Relaxation Calculated
Using Ab Initio Molecular Dynamics: Group 1 and Group 17 Ions in Aqueous
Solution. J. Chem. Theory Comput..

[39] Chubak I., Scalfi L., Carof A., Rotenberg B. (2021). NMR Relaxation
Rates of Quadrupolar Aqueous Ions from Classical Molecular Dynamics
Using Force-Field Specific Sternheimer Factors. J. Chem. Theory Comput..

[40] Campion C., Li W., Lucht B. (2005). Thermal Decomposition
of LiPF_6_ Based Electrolytes
for Lithium-Ion Batteries. J. Electrochem. Soc..

[41] Yang H., Zhuang G. V., Ross P. N. (2006). Thermal
stability of LiPF6 salt and
Li-ion battery electrolytes containing LiPF6. J. Power Sources.

[42] Schultz C., Vedder S., Streipert B., Winter M., Nowak S. (2017). Quantitative
investigation of the decomposition of organic lithium ion battery
electrolytes with LC-MS/MS. RSC Adv..

[43] Sloop S. E., Pugh J. K., Wang S., Kerr J. B., Kinoshita K. (2001). Chemical Reactivity
of PF 5 and LiPF6 in Ethylene Carbonate/Dimethyl Carbonate Solutions. Electrochem. Solid-State Lett..

[44] Wiemers-Meyer S., Winter M., Nowak S. (2016). Mechanistic insights into lithium
ion battery electrolyte degradation – a quantitative NMR study. Phys. Chem. Chem. Phys..

[45] Kawamura T., Okada S., Yamaki J.-I. (2006). Decomposition reaction of LiPF6-based
electrolytes for lithium ion cells. J. Power
Sources.

[46] Zhao K., Wan X., Lin Y., Wu H., Tan X., Zou S., Zhu M., Liu J. (2025). Magnetic Field-Based Non-Destructive Testing Techniques
for Battery Diagnostics. Adv. Energy Mater..

[47] Barskiy D. A., Blanchard J. W., Budker D., Eills J., Pustelny S., Sheberstov K. F., Tayler M. C. D., Trabesinger A. H. (2025). Zero- to
ultralow-field nuclear magnetic resonance. Prog.
Nucl. Magn. Reson. Spectrosc..

[48] Uchida S., Kiyobayashi T. (2021). How does the solvent composition influence the transport
properties of electrolyte solutions? LiPF 6 and LiFSA in EC and DMC
binary solvent. Phys. Chem. Chem. Phys..

[49] Borodin O., Smith G. D. (2009). Quantum Chemistry
and Molecular Dynamics Simulation
Study of Dimethyl Carbonate: Ethylene Carbonate Electrolytes Doped
with LiPF6. J. Phys. Chem. B.

[50] Shkrob I. A., Zhu Y., Marin T. W., Abraham D. (2013). Reduction of Carbonate Electrolytes
and the Formation of Solid-Electrolyte Interface (SEI) in Lithium-Ion
Batteries. 1. Spectroscopic Observations of Radical Intermediates
Generated in One-Electron Reduction of Carbonates. J. Phys. Chem. C.

