# Supplementary material for: Lithium Nuclear Spin Polarization Lifetimes as Sensitive Reporters of Battery Electrolyte Degradation
Source: ACS Omega. 2026 May 14;11(20):30208–13. doi: 10.1021/acsomega.6c04272 (PMC13216973; doi:10.1021/acsomega.6c04272)
Supplement: Supplementary file 1 [file ao6c04272_si_001.pdf]

Supporting Information: Lithium Nuclear Spin Polarization Lifetimes as Sensitive Reporters of Battery Electrolyte Degradation

Florin Teleanu¶,†,‡ Zhiyuan Gao¶,† and Alexej Jerschow*,†

†*Department of Chemistry, New York University, New York, NY 10003, United States*

‡*ELI-NP, "Horia Hulubei" National Institute for Physics and Nuclear Engineering, 30 Reactorului Street, Bucharest-Magurele, 077125, Ilfov, Romania*

E-mail: alexej.jerschow@nyu.edu

Composition of MD simulation boxes

As described in the main text, we set up our MD simulations boxes to mimic a 1M $LiPF_6$ solution in binary EC:DMC solvent mixtures. We tuned the number of EC and DMC molecules in order to obtain [DMC]:[EC] ratios in the range of the experimental values going from 0.78 (fresh sample) down to 0.41 (See Table S1). As initially no significant in change in ^{7}Li R_1 was predicted, we turned our attention to carbonate anions CO_3^{2-} which we added to our MD boxes. For this modification, we explored two scenarios where we added either one or two carbonate anions CO_3^{2-} for each 20 DMC molecules removed from the box (See Table S2). At the same time, we removed a corresponding number of PF_6^- anion species in order to preserve charge neutrality.

Table S1: Composition data for MD simulations of 1M $LiPF_6$ with variable ratios of [DMC]:[EC] (no CO_3^{2-} added).

#Li^+	#PF_6^-	#EC	#DMC	[DMC]:[EC]
308	308	2310	1814	0.78
308	308	2460	1664	0.67
308	308	2610	1514	0.58
308	308	2760	1364	0.49
308	308	2910	1214	0.41

Table S2: Composition data for MD simulations of 1M $LiPF_6$ for variable ratios of [DMC]:[EC] (+1(2) CO_3^{2-}/-20 DMC molecules).

#Li^+	#PF_6^-	#EC	#DMC	#CO_3^{2-}
308	308	2310	1814	–
308	294(278)	2460	1664	7(15)
308	278(248)	2610	1514	15(30)
308	264(218)	2760	1364	22(45)
308	248(188)	2910	1214	30(60)

Comparative analysis of simulated MD scenarios

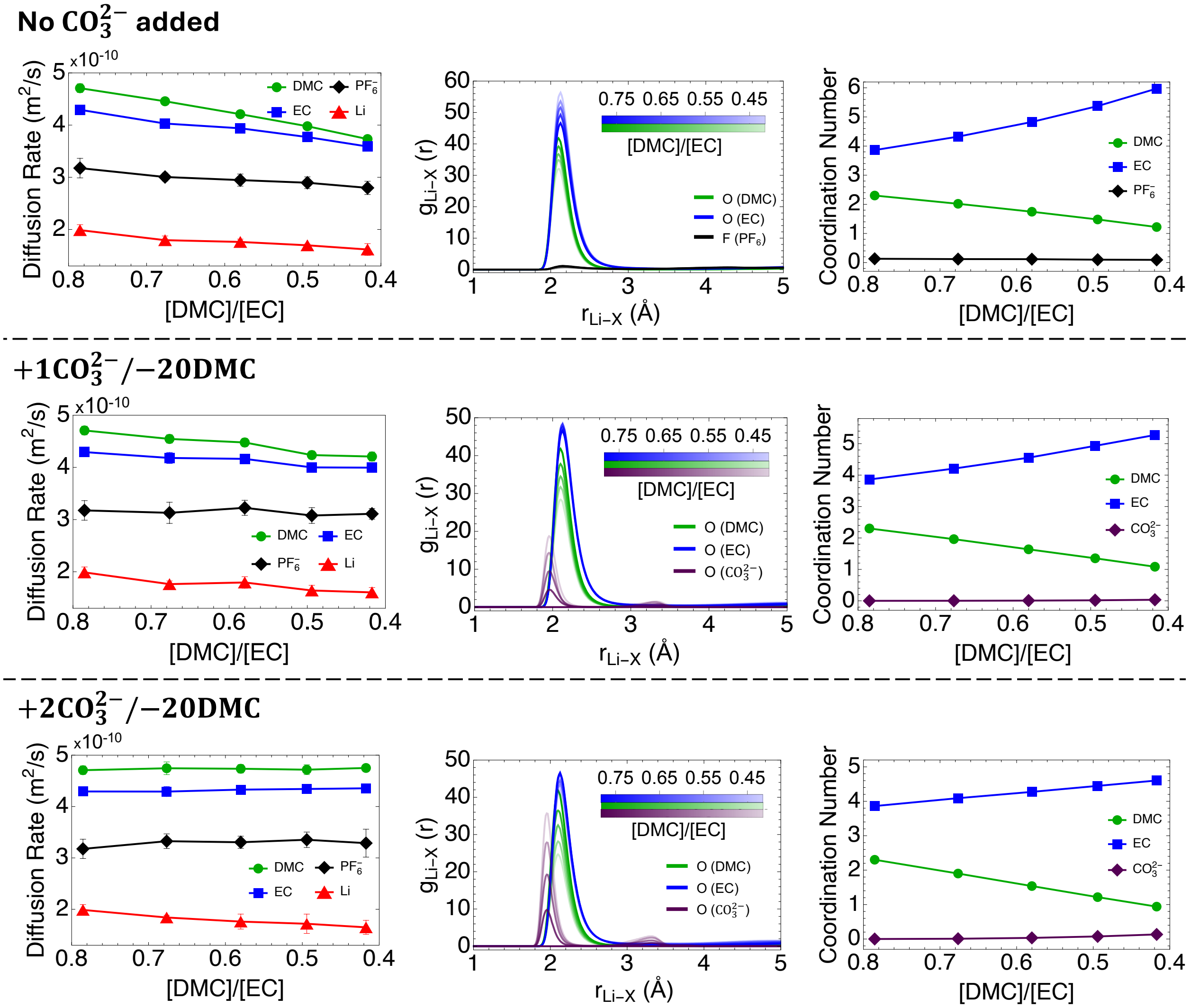

Figure S1: Molecular dynamics analysis of changes in static and dynamic properties with variable [DMC]:[EC] ratios and increasing amount (top to bottom) of carbonate anions CO_3^{2-}. (Left) Diffusion rates of electrolyte components upon decreasing [DMC]:[EC] solvent ratio. (Middle) Radial distribution functions of selected atoms around lithium ions ($g_{\text{Li-X}}$). (Right) Coordination number of selected species around lithium ions. All reported properties were derived after averaging over five independent runs (errorbars shown).
